# Accessory renal arteries in prenatal hydronephrosis as a pathophysiologic contributor or incidental vascular finding: a prospective observational study

**DOI:** 10.1186/s12884-026-09169-z

**Published:** 2026-04-29

**Authors:** Elif Ayan Avcı, Gül Alkan Bülbül, Büşra Tsakır, Hasan Berkan Sayal, And Yavuz

**Affiliations:** ¹Department of Perinatology, Antalya Training and Research Hospital, Antalya, Turkey

**Keywords:** Hydronephrosis, Accessory renal artery, Prenatal ultrasound, Doppler ultrasound

## Abstract

**Background:**

Accessory renal arteries (ARAs) are recognized as an extrinsic cause of hydronephrosis in children; however, their relationship with prenatally detected hydronephrosis remains unclear. This prospective study aimed to evaluate the prevalence and Doppler characteristics of ARAs in fetuses with antenatal hydronephrosis and to compare renal morphologic and vascular parameters with those of a control group.

**Methods:**

This prospective observational study included 123 singleton pregnancies between 18 and 36 weeks’ gestation. Fetuses were classified into a hydronephrosis group (*n* = 67) and a control group with normal renal anatomy (*n* = 56). Renal morphologic measurements and standardized renal vascular Doppler examinations were performed in all cases. The presence of ARAs was assessed using color and spectral Doppler imaging.

**Results:**

ARAs were more frequently detected in the hydronephrosis group than in controls (10.4% vs. 3.6%), although the difference was not statistically significant (*p* = 0.180). No significant differences were found in renal pelvic anteroposterior diameters (right: *p* = 0.42; left: *p* = 0.39), renal volumes (right: *p* = 0.67; left: *p* = 0.71), or main renal artery pulsatility indices (right: *p* = 0.332; left: *p* = 0.258), between fetuses with and without ARAs in either the hydronephrosis or control groups.

**Conclusions:**

ARAs were more frequently observed in fetuses with hydronephrosis than in controls; however, this difference did not reach statistical significance. Furthermore, no significant differences were observed in renal morphology or main renal artery hemodynamic parameters according to ARA status during fetal life. These findings suggest that ARAs may represent a variant of renal vascular anatomy rather than a pathological burden in the prenatal setting.

## Background

Accessory renal arteries (ARAs) represent the most common renal vascular variation and arise from the persistence of embryonic mesonephric arteries during renal ascent [1, 2]. Although renal vascular anatomy has been widely investigated in postnatal and cadaveric studies, evidence regarding ARAs in the prenatal period remains limited [3–5].

Fetal hydronephrosis is the most frequently detected urinary tract abnormality on prenatal ultrasonography, affecting 1–4% of pregnancies and occurring more commonly in male fetuses [6]. Although many cases are transient or physiological, a substantial proportion is associated with underlying pathology, most commonly ureteropelvic junction (UPJ) obstruction [7]. While UPJ obstruction is traditionally attributed to intrinsic narrowing or impaired ureteral peristalsis, the role of extrinsic factors remains controversial. Crossing accessory renal vessels have been implicated in up to 18% of postnatal hydronephrosis cases [6, 8]. Current guidelines for prenatal hydronephrosis primarily focus on morphological parameters, whereas routine evaluation of fetal renal vascular anatomy is not included in standard protocols [9–11]. Whether prenatal hydronephrosis reflects an underlying vascular phenotype, therefore, remains unclear.

This prospective study aimed to evaluate the prevalence, anatomical distribution, and Doppler characteristics of accessory renal arteries in fetuses with antenatal hydronephrosis and to compare these findings with renal morphologic measurements and a control population, thereby exploring the potential role of vascular factors in its pathophysiology.

## Materials and methods

### Study design and population

This prospective observational study was conducted at the Perinatology Clinic of Antalya Training and Research Hospital -a tertiary care center in Antalya, Turkey- between July 2025 and January 2026. The study was conducted in accordance with the Declaration of Helsinki and approved by the Antalya Training and Research Hospital Clinical Research Ethics Committee (Approval No: 10–34, Date: 19 June 2025). Consecutive eligible pregnancies were prospectively recruited, and written informed consent was obtained from all participants. This study was designed and reported in accordance with the STROBE (Strengthening the Reporting of Observational Studies in Epidemiology) guidelines. Sixty-seven singleton pregnancies diagnosed with fetal hydronephrosis between 18 and 36 weeks of gestation were included in the study. Additionally, 56 singleton pregnancies without hydronephrosis, selected to be comparable in terms of mean gestational age, were enrolled as a control group. The study was not designed as a matched cohort, and no individual matching was performed.

Participants were followed throughout pregnancy, and postnatal outcome data were collected. Pregnancies complicated by multiple gestations, maternal renal disease, or pre-existing type 1 diabetes were excluded due to their potential effects on embryonic development and vascular formation [12]. Additionally, fetuses with known chromosomal or genetic abnormalities, multiple structural anomalies, and major renal anomalies other than hydronephrosis (e.g., multicystic dysplastic kidney, pelvic kidney) were excluded to ensure a cohort of isolated hydronephrosis.

Antenatal hydronephrosis was diagnosed as dilatation of the renal collecting system on the standard transverse abdominal view, with normal ureters and bladder. Severity was classified according to the anteroposterior diameter (APD) of the renal pelvis, in line with the Fetal Medicine Foundation criteria: mild (4–7 mm in the second trimester, 7–9 mm in the third), moderate (8–10 mm, 10–15 mm), and severe (> 10 mm, > 15 mm) [9].

According to these criteria, maternal and fetal characteristics—including maternal age, gravidity, parity, gestational age, body mass index (BMI), obstetric history, fetal sex, type and anatomical side of the renal anomaly, renal pelvic diameter, renal volumes, and the presence, number, and location of accessory renal arteries (ARAs)—were documented for each case. ARAs were categorized according to their site of entry into the renal parenchyma as hilar, upper polar, or lower polar. Doppler indices were systematically recorded using standardized case report forms. Postnatal outcome data were collected for fetuses diagnosed with prenatal hydronephrosis through review of neonatal medical records and pediatric urology evaluations. Follow-up information obtained within the early postnatal period (> 48 h after birth) included the presence of spontaneous resolution or persistence of hydronephrosis and the diagnosis of underlying urinary tract pathology, such as ureteropelvic junction obstruction or vesicoureteral reflux.

### Ultrasonographic assessment

All ultrasonographic examinations were performed using a high-resolution ultrasound system (Samsung V8, Samsung Medison, Seoul, South Korea) equipped with a 2–8 MHz convex transducer, in accordance with a predefined standardized protocol. The examinations were performed by a perinatologist with more than five years of high-risk obstetric ultrasound experience using a standardized imaging protocol. Fetal kidneys were evaluated in transverse and longitudinal planes using two-dimensional grayscale ultrasonography. Renal position, contours, parenchymal echogenicity, and renal pelvic diameters were recorded. Renal length, width, and depth measurements were obtained, and renal volumes were calculated using the ellipsoid formula (length × width × depth × 0.523). To optimize image quality, maternal positioning was adjusted when necessary, with preference given to fetal positions in which the fetal back was oriented posteriorly.

### Renal vascular doppler assessment

Following grayscale evaluation, color Doppler ultrasonography was used to assess the renal vasculature. Subsequently, the abdominal aorta was visualized in a longitudinal parasagittal plane, allowing identification of the origins of the bilateral main renal arteries. To enhance visualization of low-velocity blood flow, color Doppler settings were optimized, with the pulse repetition frequency (PRF) maintained between 1.0 and 2.0 kHz and the wall filter set to 30–60 Hz. In addition, the angle of insonation was adjusted to remain below 30°. Spectral Doppler was subsequently used to confirm renal arterial flow patterns; the sample volume was set at 1–2 mm, and the velocity scale was adjusted to 20–40 cm/s. All Doppler examinations were performed in accordance with the ALARA (As Low As Reasonably Achievable) principle, with thermal and mechanical index values maintained below 1.0 throughout the examination. An ARA was defined as an arterial vessel with an independent origin, arising separately from the aorta or iliac artery and entering the renal hilus independently of the main renal artery. Blood flow within the accessory renal artery was confirmed using color Doppler imaging. Pulsatility index (PI) measurements were obtained for both the main renal artery and the ARAs. For each fetus, imaging and Doppler measurements, including PI, were repeated at least twice within the same session to ensure measurement consistency. In addition, the assessment of ARAs was not blinded to the hydronephrosis status.

To assess interobserver reliability, a randomly selected subset of 20% of cases was independently re-evaluated by a second observer. Intraclass correlation coefficients (ICC) were calculated for renal pelvic anteroposterior diameter, renal volume, and main renal artery PI measurements using a two-way random-effects model with absolute agreement (ICC [2,1]). The ICC values were 0.85 for renal pelvic anteroposterior diameter, 0.78 for renal volume, and 0.80 for main renal artery PI, indicating good interobserver agreement. In addition, the presence of accessory renal arteries (ARAs), recorded as a binary variable, was independently assessed by two observers, with complete agreement observed in all re-evaluated cases.

### Statistical analysis

The distribution of continuous variables was assessed, and data were presented as mean ± standard deviation or median (interquartile range), as appropriate. Categorical variables were expressed as counts and percentages (%). Between-group comparisons were performed using Student’s *t* test for normally distributed continuous variables and the Mann–Whitney *U* test for non-normally distributed variables. Categorical variables were compared using the chi-square test or Fisher’s exact test, as appropriate. A *p-value* of less than 0.05 was considered statistically significant. Based on a priori power analysis, the sample size was determined to provide an estimated statistical power of 88%.

## Results

**Fig. 1:**
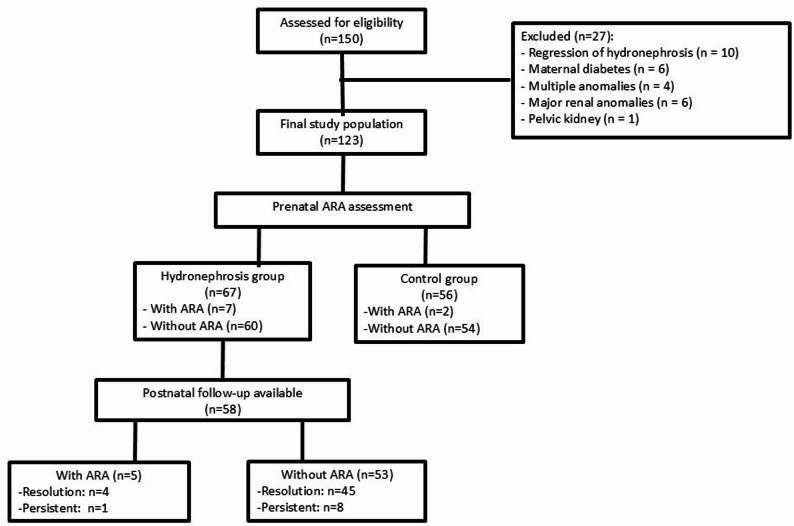
Flow diagram of the study population

A total of 150 pregnant women with singleton pregnancies between 18 and 36 weeks of gestation were initially enrolled. A total of 27 cases were excluded, including 10 fetuses with regressed hydronephrosis, 6 cases with maternal diabetes, 4 cases with multiple fetal anomalies, 6 cases with major renal anomalies, and 1 case with a pelvic kidney. Consequently, the final study population consisted of 123 fetuses: 67 with antenatal hydronephrosis and 56 controls with normal renal anatomy (Fig. 1). Among the hydronephrosis cases, 40 (59.7%) were classified as mild, 19 (28.4%) as moderate, and 8 (11.9%) as severe. Baseline demographic and obstetric characteristics were similar between the groups (Table 1), with no significant differences in maternal age, gestational age, BMI, gravidity, parity, and fetal sex distribution (all p values > 0.05).

**Table 1:** Demographic and obstetric characteristics of the study groups

Variable	Hydronephrosis (*n* = 67)	Control (*n* = 56)	*p*-value
Maternal age (years)	29.75 ± 5.36	28.70 ± 5.55	0.246
Gestational age (weeks)	27.59 ± 5.33	27.67 ± 4.89	0.792
Body mass index (kg/m²)	28.02 ± 4.55	28.69 ± 4.41	0.264
Gravidity	2.12 ± 1.41	1.96 ± 1.21	0.552
Parity	0.94 ± 1.20	0.71 ± 0.91	0.430
Male fetus	40 (59.7%)	32 (57.1%)	0.792
Female fetus	27 (40.3%)	24 (42.9%)	

Values are presented as mean ± standard deviation. Between-group comparisons were performed using Student’s t-test or chi-square test, as appropriate

Groups showed no statistically significant difference

**Fig. 2:**
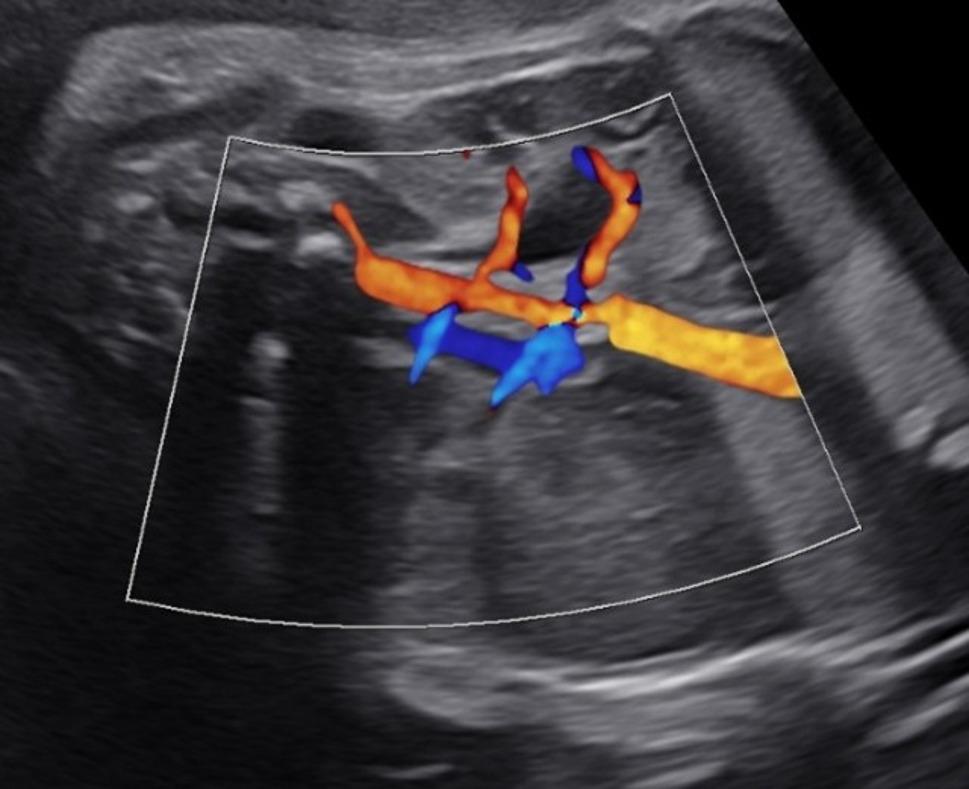
Color Doppler ultrasonographic image of the fetal kidney at 34 weeks of gestation, demonstrating bilateral ARAs extending to the renal hilar region independently of the main renal arteries

**Fig. 3:**
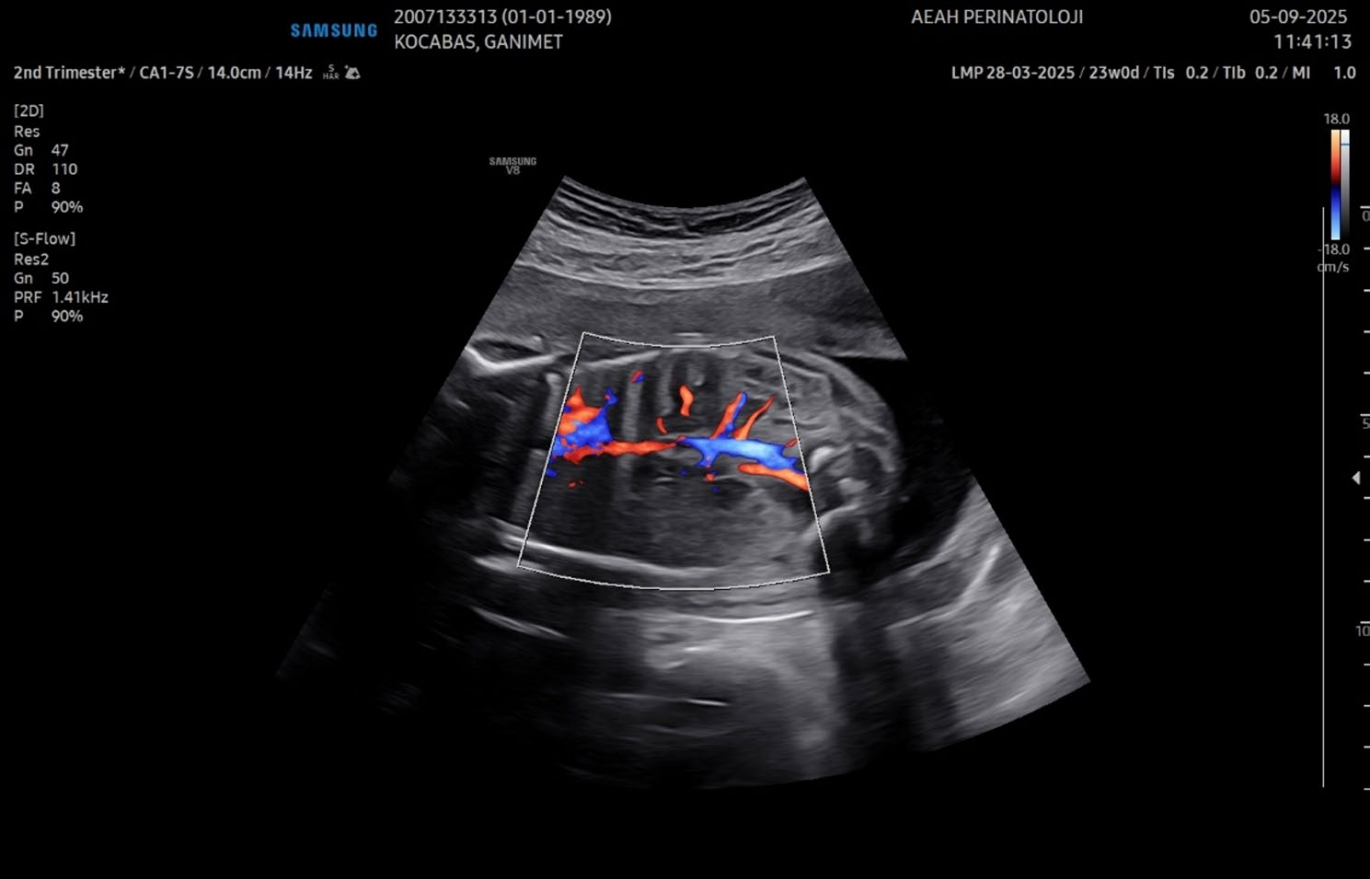
Color Doppler ultrasonographic image of the fetal kidney at 23 weeks of gestation, demonstrating a unilateral hilar accessory renal artery with an independent main renal artery

When the presence of ARAs was evaluated, they were more frequently detected in fetuses with hydronephrosis than in controls (10.4% vs. 3.6%); however, the difference did not reach statistical significance (*p* = 0.180). Within the hydronephrosis group, ARAs were identified in 7 fetuses, including 3 cases of bilateral involvement and 4 cases of unilateral involvement. In the control group, unilateral ARAs were detected in 2 fetuses, and no bilateral ARAs were observed (Table 2). When categorized by parenchymal entry site, the majority of ARAs in the hydronephrosis group were hilar (6/7), with one unilateral case demonstrating arterial entry into the lower renal pole. Notably, all ARAs detected in the control group showed a hilar entry pattern. Examples of bilateral and unilateral ARAs in fetuses with hydronephrosis at 34 and 23 weeks of gestation are shown in Figs. 2 and 3.

**Table 2:** Prevalence of accessory renal arteries (ARAs) in the hydronephrosis and control groups

Presence of ARA	Hydronephrosis (*n* = 67)	Control (*n* = 56)	*p*-value
Bilateral	3	0	
Unilateral	4	2	
ARA present*	7 (10.4%)	2 (3.6%)	0.180
ARA absent	60	54	

* Values are presented as a number (%). Between-group comparisons were performed using Fisher’s exact test

*ARA* Accessory renal artery

Renal pelvic anteroposterior diameter and renal volume were compared according to the presence of ARAs in both the hydronephrosis and control groups (Table 3). In the hydronephrosis group, no significant differences were observed in right or left renal pelvic diameters between fetuses with and without ARAs (*p* = 0.42 and *p* = 0.39, respectively). Similarly, no significant differences were observed in right or left renal volumes (*p* = 0.67 and *p* = 0.71, respectively). Comparable findings were obtained in the control group, where renal pelvic AP diameters and renal volumes did not differ according to ARA status (all p values > 0.05).

**Table 3:** Comparison of renal pelvic anteroposterior diameter and renal volume according to the presence of accessory renal arteries in the hydronephrosis and control groups

Measurement	Hydronephrosis with ARA (*n* = 7)	Hydronephrosis without ARA (*n* = 60)	*p*-value	Control ARA (+) (*n* = 2)	Control ARA (–) (*n* = 54)	*p*-value
Right renal pelvic AP diameter (mm)	8.10 ± 4.85	6.72 ± 4.19	0.42	3.10 ± 1.34	2.63 ± 1.15	0.51
Left renal pelvic AP diameter (mm)	7.54 ± 3.98	5.98 ± 3.36	0.39	3.00 ± 1.41	2.62 ± 1.12	0.58
Right renal volume (mm³)	24.8 ± 6.1	23.9 ± 5.8	0.67	23.6 ± 5.4	23.2 ± 5.1	0.74
Left renal volume (mm³)	25.1 ± 6.4	24.3 ± 5.9	0.71	24.0 ± 5.6	23.5 ± 5.3	0.69

Values are presented as mean ± standard deviation. Between-group comparisons were performed using the Mann–Whitney U test

*ARA* Accessory renal artery, *AP* anteroposterior

**Fig. 4:**
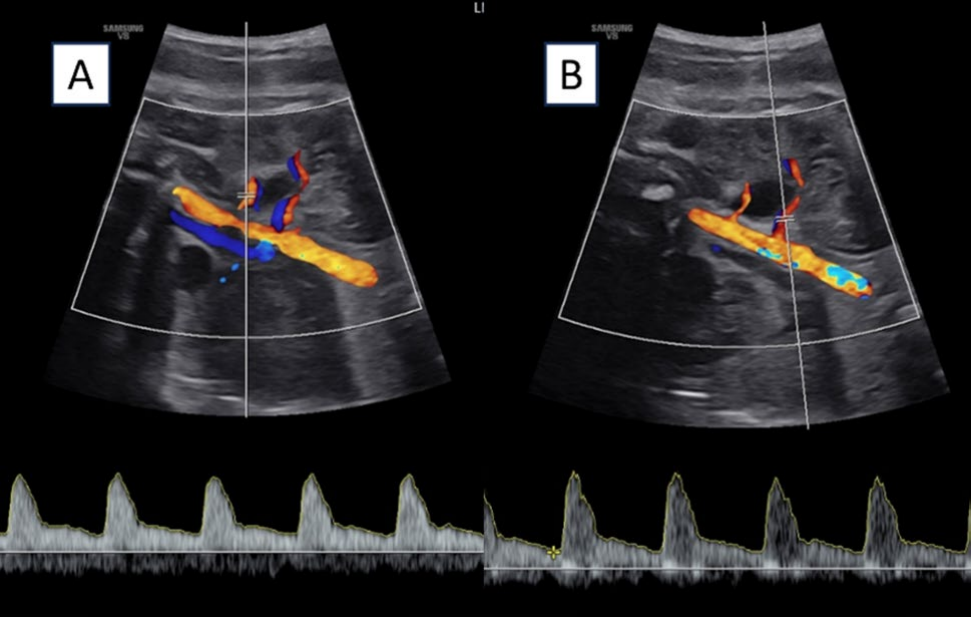
Color Doppler ultrasonographic demonstration of two distinct renal arteries supplying the fetal kidney (main and accessory) at 34 weeks of gestation (**A**), with corresponding spectral Doppler waveforms obtained separately from each vessel (**B**). Both arteries exhibit comparable and normal flow patterns

Main renal artery pulsatility indices were compared according to ARA status in both the hydronephrosis and control groups (Table 4). In the hydronephrosis group, right and left main renal artery PI did not differ significantly between fetuses with and without ARAs (*p* = 0.332 and *p* = 0.258, respectively). Similar findings were observed in the control group, where PI measurements were comparable regardless of ARA status (both *p* > 0.05). An example of a fetus with hydronephrosis demonstrating separate main and ARAs with corresponding spectral Doppler waveforms is shown in Fig. 4.

**Table 4:** Comparison of main renal artery pulsatility indices according to the presence of accessory renal arteries in the hydronephrosis and control groups

Doppler parameter	Hydronephrosis with ARA		Hydronephrosis without ARA	*p*†		Control with ARA	Control without ARA	*p*†
Right main renal artery PI		2.24 ± 0.34	2.06 ± 0.40		0.332	2.30 ± 0.14	2.17 ± 0.43	0.739
Left main renal artery PI		1.94 ± 0.45	2.22 ± 0.54		0.258	2.25 ± 0.35	2.20 ± 0.49	0.773

Values are presented as mean ± standard deviation. Between-group comparisons were performed using the Mann–Whitney U test

*ARA* Accessory renal artery, *PI* pulsatility index

Postnatal ultrasonographic follow-up performed after the first 48 h of life was available for 58 of the 67 cases (Table 5). Among these, five infants were in the group with ARAs and 53 in the group without ARAs. In the group with ARAs, spontaneous resolution of hydronephrosis was observed in 4 of 5 cases (80.0%), while persistent hydronephrosis was present in 1 case (20.0%). In the group without ARAs, spontaneous resolution occurred in 45 of 53 cases (84.9%), whereas persistent hydronephrosis was observed in 8 cases (15.1%). Among these, six cases were attributed to ureteropelvic junction (UPJ) obstruction and two to vesicoureteral reflux (VUR). Due to the small number of cases in the group with ARAs, no formal statistical comparison was performed for postnatal outcomes.

**Table 5:** Postnatal outcomes of fetuses with antenatal hydronephrosis according to ARA status

	Hydronephrosis with ARA	Hydronephrosis without ARA
Postnatal USG performed (> 48 h), *n*	5	53
Spontaneous resolution, *n (*%*)*	4 (80%)	45 (84.9%)
Persistent hydronephrosis, *n (*%*)*	1 (20%)	8 (15.1%)

*ARA* Accessory renal artery, *h* hour

## Discussion

In this prospective study, the prevalence of ARAs among fetuses with antenatal hydronephrosis was evaluated and compared with renal Doppler and morphologic parameters in a control population. The principal findings of our study indicate that although ARAs were more frequently observed in fetuses with hydronephrosis than in controls; however, the difference did not reach statistical significance. Moreover, no significant differences were identified in renal pelvic diameters, renal volumes, or renal arterial Doppler indices according to ARA status in either the hydronephrosis or control groups.

The definitive metanephric kidney develops in the sacral region in humans and receives its blood supply from different vascular sources as it ascends to its normal anatomical position. During this process, new arterial branches arise from the abdominal aorta while the lower vessels regress and disappear. This developmental sequence explains the variations observed in the number, origin, and branching patterns of the renal arteries [4]. The most common variation in renal vascular anatomy is the presence of multiple renal arteries, with an estimated incidence of approximately 21% [5]. Although ARAs have been extensively investigated in adult populations, evidence regarding their occurrence in fetal life is scarce and primarily based on cadaveric observations, leaving their prenatal clinical significance insufficiently defined [13, 14].

In one of the few prenatal ultrasonographic studies evaluating fetal renal arterial anatomy, Degani et al. reported that a single renal artery was present in 69% of fetuses, whereas vascular variations were observed in approximately 31% of cases. ARAs and arterial branching patterns were more frequently identified in fetuses with renal malformations, underscoring the clinical relevance of prenatal vascular assessment [15]. Similarly, a fetal dissection study reported accessory renal arteries in 30% of fetuses, with associated congenital renal anomalies such as horseshoe and bilateral ectopic kidneys observed in a minority of cases, suggesting a possible link between vascular variants and abnormal renal development. However, in our cohort of fetuses with antenatal hydronephrosis, no statistically significant association was found between ARAs and hydronephrosis.

In our study, the presence of an ARA was not associated with significant alterations in renal size or main renal artery Doppler parameters, suggesting a limited hemodynamic impact of these vascular variants during fetal life. Furthermore, most antenatally detected cases of hydronephrosis in fetuses with ARAs showed spontaneous resolution during the neonatal period, suggesting the notion that these findings may often represent transient or physiologic conditions. Taken together, these findings are consistent with postnatal studies reporting that hydronephrosis is frequently not detected antenatally in children with ARA–related UPJ obstruction, likely reflecting developmental characteristics of the pediatric kidney. At birth, lower pole vessels typically course cranially to the ureteropelvic junction and do not exert significant compression; however, progressive renal growth may lead to caudal displacement of these vessels and subsequent obstruction later in life [16]. In this context, given the lack of statistically significant findings and the small number of ARA-positive cases with available postnatal follow-up data, the role of these findings in guiding postnatal follow-up remains uncertain, and further large-scale prospective studies are needed to clarify this issue.

ARAs may vary in number from one to five and typically originate from either the abdominal aorta or the main renal artery, although less frequent origins such as the common iliac artery or celiac trunk have also been reported. Based on the renal territory supplied, ARAs are classified as superior polar, inferior polar, or hilar arteries [17]. Fetal studies have demonstrated substantial variability in the entry patterns of ARAs. Tom et al. reported hilar arteries in 14% of cases and polar arteries in 16%, whereas Çiçekcibaşı et al. observed comparable rates, with hilar arteries in 11.1% and polar arteries in approximately 14% of fetuses [4, 14]. In contrast, our cohort showed a clear predominance of hilar vessels, with only a single case demonstrating inferior parenchymal entry. This discrepancy may largely be explained by the inherent limitations of prenatal ultrasonography, as hilar arteries are typically larger and more centrally located, making them easier to visualize, whereas smaller polar branches may be more challenging to detect sonographically.

Previous studies have consistently reported a higher prevalence of antenatal hydronephrosis in male fetuses, with male rates often exceeding 60% and in some cases approaching 70–75% [18, 19]. However, in our cohort, fetal sex distribution did not differ significantly between the hydronephrosis and control groups. This finding may be explained by the relatively small sample size and the characteristics of our study population, particularly the low prevalence of obstructive etiologies such as ureteropelvic junction obstruction and posterior urethral valves, which are more common in male fetuses [20].

This study has several limitations. The relatively small number of ARA-positive cases may have limited the statistical power, particularly in subgroup and paired analyses, thereby increasing the risk of a type II error. Accordingly, the absence of statistically significant differences does not exclude the possibility of a clinically meaningful association. The number of ARA-positive cases with available postnatal follow-up data was very limited, which restricts the robustness of postnatal outcome analysis and warrants cautious interpretation of these findings. Furthermore, long-term renal functional outcomes were not evaluated in the present study. In addition, all ultrasonographic examinations were performed by a single experienced operator, which, while enhancing measurement consistency, may limit the generalizability of the findings. The evaluation of ARAs was performed by the same operator without blinding to hydronephrosis status. This may have introduced observer bias, as knowledge of the clinical condition could have influenced the detection of ARAs, particularly given the operator-dependent nature of Doppler ultrasonography. Moreover, prenatal detection of ARAs using Doppler ultrasonography may be technically challenging, particularly for smaller polar branches; therefore, the presence of small ARAs may have been underestimated. Finally, the study design does not represent a true matched cohort. Although the groups were comparable in terms of mean gestational age, no individual matching was performed; therefore residual confounding cannot be excluded. Nevertheless, the strengths of this study include the a priori power-based determination of sample size, the prospective study design, the use of a standardized Doppler protocol, and the simultaneous evaluation of both main and ARAs.

In conclusion, the present study demonstrates that ARAs are more frequently observed in fetuses with hydronephrosis than in controls; however, this difference did not reach statistical significance. Furthermore, these vascular variations appear not to significantly affect renal pelvic dilatation, renal volume, or main renal artery hemodynamics during the prenatal period. These findings suggest that ARAs may represent a variant of renal vascular anatomy rather than a pathological burden in the prenatal setting. These findings provide a basis for future prospective and long-term studies aimed at elucidating the role of vascular factors in the evaluation and pathophysiology of prenatal hydronephrosis.

## Data Availability

The data that support the findings of this study are available from the corresponding author upon reasonable request.

## Abbreviations

ALARA: As Low As Reasonably Achievable
AP: Anteroposterior
ARA: Accessory Renal Artery
BMI: Body Mass Index
ICC: Intraclass Correlation Coefficient
PI: Pulsatility Index
PRF: Pulse Repetition Frequency
STROBE: Strengthening the Reporting of Observational Studies in Epidemiology
UPJ: Ureteropelvic Junction
USG: Ultrasonography
